# One-Stop Dispensing: Hospital Costs and Patient Perspectives on Self-Management of Medication

**DOI:** 10.3390/pharmacy6020046

**Published:** 2018-05-28

**Authors:** Morten Baltzer Houlind, Helle Bach Ølgaard McNulty, Charlotte Treldal, Signe Lindgaard Andersen, Thomas Huneck Haupt, Janne Petersen, Ove Andersen, Lene Juel Kjeldsen

**Affiliations:** 1 Optimed, Clinical Research Centre, Copenhagen University Hospital Hvidovre, Kettegård Alle 30, Department 056, 2650 Hvidovre, Denmark; charlotte.treldal.02@regionh.dk (C.T.); signe.lindegaard.andersen@regionh.dk (S.L.A.); thomas.huneck.haupt.01@regionh.dk (T.H.H.); janne.petersen.01@regionh.dk (J.P.); ove.andersen@regionh.dk (O.A.); 2 The Capital Region Pharmacy, Marielundvej 25, 2730 Herlev, Denmark; helle.bach.oelgaard.mcnulty@regionh.dk; 3 Section of Biostatistics, Department of Public Health, University of Copenhagen, 1014 Copenhagen, Denmark; 4 Emergency Department, Copenhagen University Hospital, 2650 Hvidovre, Denmark; 5 Amgros I/S, Dampfærgevej 27, 2100 Copenhagen, Denmark; ljuelkjeldsen@gmail.com

**Keywords:** One-Stop Dispensing, medication systems, hospital, self administration, self-management, patient medication knowledge, patient satisfaction, pharmacy service, hospital, workload, medication, inpatient, hospitalization

## Abstract

(1) Objective: To assess hospital medication costs and staff time between One-Stop Dispensing (OSD) and the Traditional Medication System (TMS), and to evaluate patient perspectives on OSD. (2) Methods: The study was conducted at Hvidovre Hospital, University of Copenhagen, Denmark in an elective gastric surgery and acute orthopedic surgery department. This study consists of three sub-studies including adult patients able to self-manage medication. In Sub-study 1, staff time used to dispense and administer medication in TMS was assessed. Medication cost and OSD staff time were collected in Sub-study 2, while patient perspectives were assessed in Sub-study 3. Medication costs with two days of discharge medication were compared between measured OSD cost and simulated TMS cost for the same patients. Measured staff time in OSD was compared to simulated staff time in TMS for the same patients. Patient satisfaction related to OSD was evaluated by a questionnaire based on a five-point Likert scale (‘very poor’ (1) to ‘very good’ (5)). (3) Results: In total, 78 elective and 70 acute OSD patients were included. Overall, there was no significant difference between OSD and TMS in medication cost per patient ($2.03 [95% CI −0.57–4.63]) (*p* = 0.131). Compared with TMS, OSD significantly reduced staff time by an average of 12 min (*p* ≤ 0.001) per patient per hospitalization. The patients’ satisfaction for OSD was high with an average score of 4.5 ± 0.7. (4) Conclusion: There were no differences in medication costs, but staff time was significantly lower in OSD and patients were overall satisfied with OSD.

## 1. Introduction

Medication errors occur in half of all hospitalized patients and can be associated with adverse drug events [1,2]. Common reasons for medication errors include incomplete medication history [3,4,5,6], mistakes when dispensing or administering medication [7,8], suboptimal discharge processes [9,10], and lack of patient medication information at discharge [11,12]. These challenges should be addressed by future medication systems, and the solutions must be feasible for both existing and newly built hospitals.

In the Traditional Medication System (TMS), medication is dispensed and administered manually by hospital staff on the wards. Medications are dispensed from large original containers into a cup and manually transported to the patient in a trolley. Various ward-based and hospital-centralized systems for packing, dispensing and administering medication are used internationally without patient involvement. Examples included computer-controlled automated dispensing cabinets [13] and automated dose dispensing, where medication is machine-packed into patient-specific bags [14]. However, new approaches aim to increase patient involvement [15,16,17], including patients as active partners [18] and self-management in the medication systems [19,20]. A common feature of these new approaches is getting patients more active in their own health and disease management. Patients are likely to know about their own medications used prior to hospitalization, and allowing patients to continue managing their own medication when hospitalized could prevent disruption of daily practices and may reduce medication errors [15,21,22]. A systematic literature review found significant improvement in patients’ medication knowledge and compliance when medication was self-managed [23]. However, findings on used staff time, patient satisfaction and medication costs are inconsistent [23].

One-Stop Dispensing (OSD) is a bedside medication system in which patient involvement is an essential component [16,17]. In OSD, patients’ own medication (POM) is used during hospitalization and the patients self-administer their medication when it is assessed to be safe. Patients are continuously informed and trained in how to manage their own medications during hospitalization and at discharge [16,17]. In the literature, this concept is known as either self-administration or self-management of medication. Vanwesemael et al. define self-management of medication as a broader range of activities including patient education and monitoring while patients self-manage their medication [19]. Based on this definition, self-management is compatible with the OSD concept. However, there is a lack of knowledge about hospital cost and patients’ perspectives on OSD for elective and acutely hospitalized patients. Furthermore, OSD has never been studied in a Danish setting. The objectives of this study were: (1) to compare hospital medication costs for OSD and TMS; (2) to compare total nurse and pharmacy staff time used to dispense medication, administer medication and speak with patients about their medication in OSD and TMS; (3) and to study patient perspectives on OSD, self-management of medication and use of POM.

## 2. Materials and Methods

### 2.1. Ethics Approval

The study was approved by the Danish Capital Region’s Data Protection Agency (j.no. RAP2014-003). Patients included in the intervention signed a written informed consent at inclusion, while the control group receiving TMS was part of routine quality assurance corresponding to a Zelen randomization procedure [24].

All observations of staff were also performed in accordance with the Helsinki Declaration. The head nurse and head pharmacist received written and verbal information about the study and approved it prior to its start. All nursing and pharmacy staff involved in the study were provided with written and verbal information during at least two staff meetings. The staff was informed that participation was voluntary and they could withdraw from the study at any time. All data was anonymized before any analysis, and no personal data about the observed staff was collected.

### 2.2. Setting

The Danish healthcare system is primarily tax funded, has universal coverage of citizens, and is based on the principles of free and equal access to health care for all citizens [25]. All medications used during hospitalization in Denmark is tax-paid. In the primary sector, annual self-payment for medicine for Danish citizens cannot exceed $650 as a result of the tax system. Amgros I/S, Copenhagen, Denmark is a company owned by Danish Regions that negotiates medicine prices with the pharmaceutical industry for all Danish hospitals. Prices are negotiated especially for frequently used medications and often only for the largest pack sizes. The Danish Health Act states that it is permitted to ask patients to use POM during hospitalization.

This study was conducted at Hvidovre Hospital, University of Copenhagen, Denmark. Hvidovre Hospital contains 650 beds and has an annual inpatient admission of 95,600. This study included patients from the elective gastric surgery (elective) ward and an acute orthopedic surgery ward (acute). The elective ward treats lower gastrointestinal diseases with surgery and medication in a 22-bed unit. The acute ward performs non-traumatic lower limb amputations, minor surgery and pain management in a 26-bed unit.

### 2.3. Design and Patients

This study consists of three sub-studies as described below:

- In Sub-study 1, data regarding staff time used to dispense and administer medication in TMS was collected. Data for the TMS group was collected from both wards in February 2015.
- In Sub-study 2, medication costs for OSD were compared to simulated TMS costs for the same patients. In a subset of Sub-study 2, staff time used in OSD was compared to simulated staff time used in TMS for the same patients. The simulations of TMS were based on results from Sub-study 1. Data was collected from April to June 2015.
- In Sub-study 3, a questionnaire was used to assess patient perspectives on OSD. Data was collected from September 2015 to November 2015.

Inclusion criterion for all three sub-studies was: age ≥ 18 years. Exclusion criteria were: inability to self-manage medication prior to admission; inability to understand Danish; decrease in cognitive, emotional or health condition during hospitalization resulting in unsafe/lacking ability to self-manage medication; history of medication abuse; terminal disease; suicidal ideation; isolation; and use of patient-specific automated packed medication prior to hospitalization (a uni-dose medication packing service sold by primary Danish pharmacies). Information about exclusion criteria was collected from patient records and patient interviews. Patients were excluded if there was any doubt about meeting the exclusion criteria.

A senior nurse or physician evaluated each patient’s current cognitive, emotional and health condition with regard to their ability to self-manage medication during hospitalization. This was performed as part of routine clinical work with the patients and completed during each staff shift (three times per 24-h). The result of the assessment was noted in the patient record. If the patient’s condition changed and the staff evaluated self-management of medication unsafe, the patient was excluded. Pharmacy staff recruited patients at both wards. Elective patients were recruited during an initial interview before hospitalization, while acute patients were recruited directly upon admission.

### 2.4. Intervention

Patient lists for both wards were screened for potential OSD participants by pharmacy staff. Elective patients were asked to bring their own medication in original containers prior to hospital admission, while acute patients were asked to bring their own medication within 24 h after admission. Upon inclusion, pharmacy staff recorded an updated medication history for all patients and conducted quality control of POM in accordance with the criteria described by Nielsen et al. [26] In case of discrepancies between the recorded medication history and the patient’s electronic medication record, a physician performed medication reconciliation. Pharmacy staff checked each patient’s ability to self-manage medication based on criteria from Edelberg et al. [27]. Patients’ ability to identify, access, dose and time their own medication was reviewed and approved by pharmacy staff.

Patients began to self-manage medication from the first day after surgery. Self-management of medications included both POM for chronic conditions and medication prescribed during hospitalization (e.g., proton pump inhibitors, analgesics, and antibiotics). Pharmacy staff distributed the smallest available original containers and resupplied POM when necessary. Each patient received a medication list including indication, dosage, and duration of treatment for each medication at day one or after any prescription changes. All medication included in OSD were stored in a lockable bedside locker accessible to patients at all times. Medications prescribed “as needed” during hospital admission or stored in a refrigerator were still administrated by the staff. The TMS group received standard care as described previously. 

#### One-Stop Dispensing from Day 2 to Discharge

Patients included in OSD were attended to by nursing or pharmacy staff on ward rounds. The patients’ medications were continually supplied, and prescriptions were updated when necessary. Containers were re-labeled to reflect changes in dose of any self-managed medications. Medication changes were systematically reviewed with patients and supported with an updated medication list. Patients were encouraged to ask questions about their medication during hospitalization. At discharge, patients received an updated medication list after a physician, in cooperation with pharmacy staff, conducted medication reconciliation. Patients were discharged with at least ten days’ supply of all current medications, including both chronic conditions and acute conditions acquired during hospitalization.

### 2.5. Data Collection

Descriptive data regarding patient characteristics was collected from patient records. This data included age, sex, number of medications before hospitalization, number of medications dispensed per day during hospitalization, and length of hospitalization.

### 2.6. Medication Cost

Pharmacy staff recorded all medications dispensed and used by patients during hospitalization. Medication costs in OSD (Sub-study 2) were calculated individually for each patient based on the hospital pharmacy’s medication prices. Medication cost in TMS was simulated for each patient. Medications in TMS normally cover 2 days after discharge, while medications in OSD can cover weeks after discharge depending on medication container size and duration of treatment [16,17]. Costs for OSD were calculated in four ways: total costs during hospitalization plus two days after discharge with and without nutritional supplements, and total costs during hospitalization plus ten days after discharge with and without nutritional supplements. Calculations were performed with ten days of discharge medication to explore the cost of switching to OSD whit this service improvement.

### 2.7. Time Measurements and Procedure for Nursing and Pharmacy Staff

Time measurements for TMS and OSD were performed with a stopwatch by an observer between 06:30 and 23:30 in Sub-study 1 and 2. In this period, all routine staff work is performed for both TMS and OSD. In the TMS group, total staff time to dispense and administer medication as well as time to dialogue with the patient about their medication was measured during five consecutive days. At the elective ward, these medication processes were always performed by nursing staff. At the acute ward, the medication processes were performed by pharmacy staff between 07:00 and 14:00 Monday to Friday and nursing staff the rest of the time. Time measurements for TMS included all medications except of “as needed” medication and intravenous formulations. Medications at the hospital were as standard given at 8:00, 12:00, 17:00 and 22:00. Staff time used to administer medication was only measured for patients who were able to self-manage medication prior to admission according to their electronic patient journal.

The OSD time measurements were collected during five consecutive days, and time measurements were followed for these patients until the day of discharge. The measurements also entailed all staff time used to dispense and administer medication and daily dialogue with the patient about their medication. The dispensing process included quality check of POM, labeling, print of medicine list and supplying bedside lockers with medication. The administration process included checking of patients’ ability to self-manage medication. At both wards, pharmacy staff was in charge of medication processes related to OSD between 08:00 and 15:00 Monday to Friday, while nursing staff covered these responsibilities at all other times. “As needed” medication and intravenous formulations were not at part of the OSD system and were not included in the measurements.

For both TMS and OSD medication was available at the wards in a medication room. Ordering and refilling the medication room was not included in the time measurements. At all time-measurements, the time was paused if staff was interrupted by events not related to the specific process. Finally, in the time measurement for both TMS and OSD, we also included nursing staff time used on medication dialogue with patients while pharmacy staff was on the ward. To adjust for variations in the number of medicines per patient, staff time used for OSD in Sub-study 2 was compared to simulated TMS time used for the same patients on an individual patient level (Equation (1)). Staff time used to dispense and administer medication in the TMS group was used in the simulations. Number of medications dispensed per day during hospitalization and length of stay was also taken into account (Equation (1)).

**Equation (1):** Calculation of differences in staff time between OSD and simulated TMS per patient.

(1) $$\begin{array}{l} \mathrm{Time}\;\mathrm{difference}\;\left(\mathrm{OSD}-\mathrm{TMS}\right)\;\mathrm{per}\;\mathrm{patient}\;\left(\min\right)\\ =({\mathrm{OSD}}_{\mathrm{start}}\left(\min\right)+\left({\hat{\mathrm{OSD}}}_{\mathrm{day}2+\;}\left(\min/\mathrm{day}\right)*\left(\;\mathrm{LOS}\;\left(\mathrm{days}\right)-1\right)\right)\\ -\;\left(N_{\mathrm{Medications}\;\mathrm{disp}./\mathrm{day}}*\;{\hat{\mathrm{TMS}}}_{\;\mathrm{time}\;\mathrm{to}\;\mathrm{disp}.\mathrm{one}\;\mathrm{medication}\;}(\min)\right)\;\\ +(N_{\mathrm{Medications}\;\mathrm{adm}./\mathrm{day}}*\;{\hat{\mathrm{TMS}}}_{\;\mathrm{time}\;\mathrm{to}\;\mathrm{adm}.\mathrm{of}\;\mathrm{medication}\;}(\min))*\mathrm{LOS}(\mathrm{days})) \end{array}$$

where OSD_start_ (min) is time in minutes used on OSD day 1 for each patient, ${\hat{\mathrm{OSD}}}_{\mathrm{day}2+\;}$ (min/day) is average daily time in minutes used for continuation of OSD from day 2 for each patient, LOS is length of stay in days, $N_{\mathrm{Medications}\;\mathrm{disp}/\mathrm{day}}$ is number of medications dispensed per day in Sub-study 2, $\;{\hat{\mathrm{TMS}}}_{\;\mathrm{time}\;\mathrm{to}\;\mathrm{disp}.\mathrm{one}\;\mathrm{medication}\;}(\min)$ is estimated time in minutes to dispense one medication using TMS in Sub-study 1, $N_{\mathrm{Medications}\;\mathrm{adm}./\mathrm{day}}$ is number of medication administrations per day in Sub-study 2, and ${\hat{\;\mathrm{TMS}}}_{\;\mathrm{time}\;\mathrm{to}\;\mathrm{one}\;\mathrm{adm}.\mathrm{of}\;\mathrm{medication}\;}(\min)$ is estimated average time in minutes for one medication administration in Sub-study 1.

### 2.8. Patient Perspectives and Satisfaction

Patient perspectives and satisfaction related to OSD were assessed by a questionnaire in Sub-study 3. The questions were related to the use of POM, bedside lockers, medication containers, medication information, responsibility of self-management, handling of medication after discharge, and ability to self-manage medication at future hospitalization.

The design of the questionnaire was based on (1) literature exploring patient perspectives on OSD, self-management of medication, and use of POM [28,29,30]; and (2) thematic analysis of 35 telephone interviews with OSD patients (unpublished feasibility study). Initially, a questionnaire with 11 items was developed. Afterwards, the questionnaire was pilot tested for validity and reliability by using a convenient sample of six patients. As a result of this first pilot test, two questions were deleted due to overlap in context, and another question was divided into two separate questions to increase clarity. Finally, number options (1 to 5) were added to questions graded on a Likert scale. After these changes, the questionnaire was pilot tested by another 6 patients. As a result of this second pilot test, grammar was corrected for one question.

At discharge, patients were asked to fill in the ten item questionnaire. A five-point Likert scale ranging from ‘very poor’ (1) to ‘very good’ (5) was used to assess eight items, while two questions could be answered with ‘yes’ or ‘no’. ‘Do not know’ was an acceptable answer for all questions.

### 2.9. Statistics

Comparison of patient characteristics was performed with a non-parametric test since not all parameters were normally distributed. Chi-square test was used for statistical comparisons between distributions of sex. Two-sided Mann-Whitney test was used to compare median values between age, number of medications before hospitalization, number of medications dispensed per day during hospitalization, and length of hospitalization.

The main outcomes of the study were the differences in medication cost and differences in staff time between OSD and TMS. This was analyzed by parametric statistics in order to maximize power. Differences in medication cost between TMS and OSD with two days of discharge medication without nutritional supplements was compared using a paired *t*-test on pooled data from elective and acute patients. All analyses were also performed individually for elective and acute patients. Staff time was compared between OSD and TMS based on pooled data from elective and acute patients. Paired *t*-test was used to compare measured staff time used in OSD and simulated staff time used in TMS for the medication dispensing process during hospitalization. Mean patient satisfaction scores for questions based on the Likert scale were compared between elective and acute patients with an unpaired t-test. Answers to Likert scale questions are presented in numbers and percentages based on pooled data from elective and acute patients. IBM SPSS Statistics 22.0 (IBM, Armonk, NY, USA) was used for all statistical analyses. A *p*-value of <0.05 (two-sided) was considered to be statistically significant.

## 3. Results

**Figure 1:**
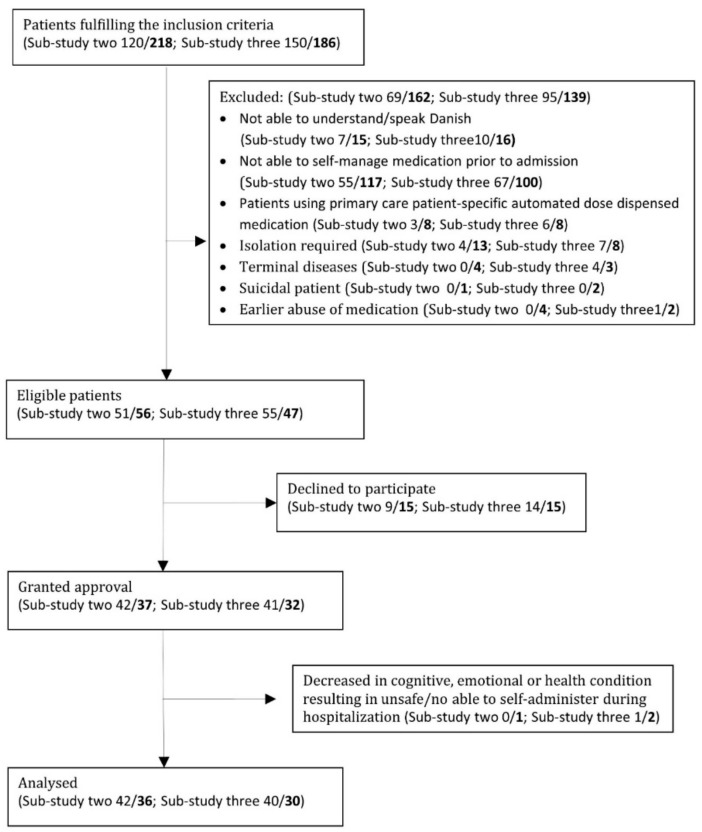
Flowchart of inclusion of OSD patients in the study (total *n* = 148). Elective patients on the left side of the slash and acute patients on the right side marked with bold font.

On inclusion days, 674 patients were admitted to the elective and acute wards. Of these, 465 patients were excluded (Figure 1). Inability to self-manage medication prior to admission was the primary reason for exclusion among both gas sur. (45%) and ort sur. (54%) patients. Of the remaining 209 eligible patients, all patients were asked to participate, and 152 consented to participation. Of these, four patients were excluded because of decrease in cognitive, emotional or health condition during hospitalization. A total of 148 patients participated in the OSD intervention—78 from the elective ward and 70 from the acute ward. Combined patient characteristics for both sub-studies are presented in Table 1.

No differences were found in OSD patient characteristics between Sub-study 2 and 3 for elective (all *p* ≥ 0.109) or acute patients (all *p* ≥ 0.118). Eighty-seven patients were included in Sub-study 1, comprised of 31 patients from the elective ward and 56 patients from the acute ward. No differences in patient characteristics between TMS and OSD were found for elective or acute patients (all *p* ≥ 0.092).

Overall, no difference (*p* = 0.13) was found in medication costs between OSD patients with two days of discharge medication without nutritional supply compared to TMS (Table 2). In OSD, there was a mean additional cost per patient of $2.03 (95% CI −0.57–4.63). Of all medications used prior to hospitalization, POMs accounted for 87% of items in elective patients’ and 67% in acute patients.

In total, 24 patients (11 elective and 13 acute) were included in the study of staff time as part of Sub-study 2. No significant differences in patient characteristics were observed for these 24 patients and the patients in Sub-study 1 (all *p* ≥ 0.067). Among the patients in Sub-study 2, total staff time used per patient was significantly lower (*p* ≤ 0.001) for OSD compared to simulated TMS (Table 3). On average, time spent per patient in OSD was 12 min lower for the combined group of elective and acute patients. There was no significant difference in time saved by OSD between elective and acute patients (*p* = 0.316). Average time spent by staff per medication dispensed in TMS was 0.70 ± 0.13 min (*n* = 651) and 0.57 ± 0.10 min (*n* = 1896) in the elective ward and acute ward, respectively. The administration process of medication in TMS took on average 0.34 ± 0.09 min (*n* = 151) and 0.27 ± 0.07 min (*n* = 281) per patient per administration in the elective ward and acute ward, respectively. Time to dispense and administer medication to each patient per day was 16.2 ± 6.61 min (*n* = 87) for the combined group, while it was 14.1 ± 6.40 (*n* = 31) min and 17.2 ± 6.72 (*n* = 56) min for elective and acute patients, respectively.

Patient perspectives related to OSD are given in Table 4. Mean score for the combined group of patients was 4.5 ± 0.7, while it was 4.4 ± 0.7 and 4.5 ± 0.7 for elective and acute patients, respectively. No difference in scores was observed between elective and acute patient (*p* = 0.07). Mean scores for the eight questionnaire items ranged from 4.2 to 4.8. In addition, 44% of patients answered that OSD had improved handling of their own medications after discharge. Eighty-two percent of patients wished to receive information about their medication from pharmacy staff in the future.

## 4. Discussion

Our study found no significant difference in medication cost for OSD with two days of discharge medication without nutritional supply compared to TMS. We found that staff time used in medication processes was significantly lower in OSD compared to TMS. Patients in OSD rated the system with an overall high average score of satisfaction and safety.

Overall, we found that hospital medication costs in OSD for 2 days of discharge medication without nutritional supply was only $2.03 (CI 95 −0.57–4.63) more per patient compared to TMS. Discharge with 10 days with medication and/or nutritional supply resulted in additional costs in OSD compared to TMS. Discharge medication for 10 days is a service improvement for patients in the Danish health system, but it is unknown whether such a service would lead to better health outcomes. Finally, we observed a tendency for an additional cost in OSD for acute patients compared to elective patients. The literature provides contradicting results about how use of patients own medication and elements of OSD affects hospital medication costs [31,32,33,34]. Possible reasons for different findings in medication costs between studies can be explained by differences in medication cost among groups of patients, different access to patients own medication or different amount of medication waste between groups’ patient and setups. Stable medication therapy results in less medication waste. In patient groups using costly medications (e.g., non-Vitamin K antagonists oral anticoagulants, glucagon-like peptide-1 receptor analogues, long-acting muscarinic receptor antagonists), hospital savings can increase by using POM. In this study, we calculated differences in medication costs for the same patients, making our results less susceptible to bias based on absolute medication cost.

We found that OSD significantly reduced staff time by an average of 12 min per patient per hospitalization. Results from other studies are inconsistent [31,35,36,37], and differences between patient groups, setups and methodology makes it difficult to compare results from other studies. We found that staff used 0.57–0.70 min to dispense one medication in TMS, which is consistent with findings by Buck et al. in a geriatric ward [38]. We also found that from day 2 of OSD to discharge, hospital staff spent an average of 5–6 min per patient per day. This result is in contrast to Grantham et al., which found that staff used 20 min per patient per day for ongoing assessment and education. However, patients in Grantham et al. were in a Nursing Convalescent Unit with mean age of 68 years, so these patients may have needed more support to handle their own medications compared with patients in our study [35].

In our setting, the POMs were provided for 87% of all prescribed medication in elective patients and 67% for acutely hospitalized patients. Nielsen et al. found that 59% of all patients in the emergency department independently brought their own medications to the hospital [26]. In the United Kingdom, a “green bag scheme” encourages all patients and ambulance staff to bring POM to the hospital [16,17]. Implementation of a similar scheme in Denmark could increase the number of medications provided by patients. Lastly, medication prices in Denmark are currently only negotiated for medications in large package sizes, but price negotiations for medications in all package sizes will support the full economic potential of the OSD intervention.

In this study, we found a high average score for patient perspectives on satisfaction, safety and responsibility related to OSD. Other studies have found similar results among patients who self-manage their medications [29,31,35,39,40]. However, our findings are in contrast to a study from the United Kingdom by Tan et al., in which 19% of participants were dissatisfied with the concept. The main reason for the dissatisfaction in that study was difficulty with bedside lockers [41]. Some patients in our study reported similar problems, with six percent of elective patients rating “poor” satisfaction with bedside lockers. 82% of patients responded “yes” to the question, “Would you want pharmacist staff in the future to inform you about your medicine during hospitalization?”. This finding supports the description in Vanwesemael et al. 2017 which suggest a multidisciplinary staff approach for maximum benefits of self-management of medications in hospital [19].

We believe that three findings from our patient questionnaire are particularly important for designing future medication systems: first, 94% of patients were satisfied with the responsibility of self-managing their own medication; second, 83–85% of patients were satisfied with receiving medication information; third, 44% of patients found the system had improved handling of their own medications after discharge. Given these patient reports, implementing OSD on hospitals wards has the potential to increase patient knowledge, compliance and quality of medication histories across sectors, as well as reduce medication errors. Taken together, our findings indicate that OSD with self-management of POM can improve patient care and reduce staff time used in medication processes, in both hospitalized patients, despite small increases in medication cost. Considering the potential costs saved by reduced staff time, OSD could potentially even reduce total hospital costs. This element is central because self-management of medication must reduce hospital costs and/or improve health outcomes in order to be used broadly in daily hospital practice. An example of improved health outcome by self-management of medication are reduced pain identity in orthopedic patients who self-controlled analgesics [42]. A recent publication by Vanwesemael et al. suggests additional strengths of patient self-management of medication, including enhanced medication adherence, better patient education, better monitoring of medication use, and reduced disruption of daily medication routines [20]. Future studies should aim to understand patient perspectives on these aspects of self-management.

The primary strength of our study is that it was conducted under daily context-specific conditions in both an acute and elective hospital ward. Time measurements from both departments show that medication dispensing and administration in the hospital is a time-consuming process. However, one limitation of our study is that we did not consider medications prescribed “as needed”, or medication given at discharge. Several studies have previously shown that OSD reduces staff time at discharge [16,35] but we did not include this analysis in our study. We also did not investigate patients’ perspectives on TMS or staff perspectives, which should both be considered in comparison to OSD. Another important limitation of the study is in our study design, in which we compare OSD in an intervention group (Sub-study 2) with TMS (Sub-study 1). Measuring at two different time periods increases the risk of biased results caused by other changes in the department in the time between the data collection time points, though we did not observe any organizational changes in the time period. Similarly, it is a limitation that OSD cost (Sub-study 2) and patient perspectives (Sub-study 3) were measured at two different time periods. Importantly, the same resources were allocated to OSD at both time periods, and we observed no changes in the OSD intervention or organization between the two periods. A fourth limitation is that our study has not been powered to show a difference in medication cost and staff time between medication systems on a ward level. Differences in hospital costs between countries due to medication pricing, subsidies and dosing guidelines should also be considered. Finally, we do not know the possible long term effects of OSD and its impact on medication errors, compliance, treatment effect and health. In the future, these outcomes must be measured in randomized controlled trials in different patient groups, particularly in patients receiving polypharmacy.

## 5. Conclusions

This study found no significant differences in medication cost per patient in OSD compared to TMS. Staff time to dispense medication, administer medication and speak with patients about their medication was significantly lower in OSD compared to TMS. Patients rated OSD with an overall high average score of satisfaction and safety. In hospital medication systems, self-management of medicine seems to have a position in the future. Future studies should investigate health-related outcomes regarding self-management of medication in hospital.

## Figures and Tables

**Table 1:** Patient characteristics, represented as median values with range (minimum-maximum).

Patient Characteristics	Sub-Study 1 TMS		Sub-Study 2 & 3 OSD		Comparison TMS and OSD
	Elective (*n* = 31)	Acute (*n* = 56)	Elective (*n* = 78)	Acute (*n* = 70)	*p*-Value Elective|Acute
Age, years *	50 (23–84)	60 (29–94)	55 (19–82)	52 (22–81)	0.231|0.092
Female sex, n (%) **	21 (68)	34 (60)	55 (71)	41 (59)	0.958|0.564
Medications before hospitalization *	3 (0–9)	4 (0–9)	3 (0–9)	3 (0–14)	0.509|0.213
Medication dispensed/day during hospitalization *	14 (7–22)	18 (4–26)	13 (6–21)	16 (6–24)	0.318|0.642
Length of stay, days *	3 (1–9)	5 (2–13)	4 (2–11)	5 (1–12)	0.219|0.368

TMS Traditional Meditation System, OSD One Stop Dispensing. * Two-sided Mann-Whitney test; ** Chi-square test.

**Table 2:** Additional medication cost per patient for OSD compared to TMS, represented as mean $ difference ± SD (95% confidence interval).

Medication Cost Comparison between OSD and TMS	Combined (*n* = 82)	Elective (*n* = 42)	Acute (*n* = 40)
During hospitalization and 2 days after discharge without nutritional supply	$2.03 ± 12.05 (−0.57; 4.63) *p* = 0.131	$1.46 ± 13.05 (−2.44;5.36) *p* = 0.473	$2.62 ± 10.18 (−0.58; 5.82) *p* = 0.112
During hospitalization and 2 days after discharge	$2.71 ± 11.59 (0.21; 5.21) *p* = 0.037	$2.31 ± 11.69 (−1.19;5.81) *p* = 0.208	$2.67 ± 11.49 (−0.93; 6.27) *p* = 0.150
During hospitalization and 10 days after discharge without nutritional supply	$3.13 ± 11.88 (0.53; 5.73) *p* = 0.019	$1.49 ± 13.05 (−2.41;5.39) *p* = 0.464	$4.86 ± 10.51 (1.38; 7.98) *p* = 0.006
During hospitalization and 10 days after discharge	$3.81 ± 11.68 (1.31; 6.31) *p* = 0.004	$2.33 ± 11.65 (−1.17;5.83) *p* = 0.202	$5.32 ± 12.04 (1.62; 9.02) *p* = 0.008

Combined (*n* = 82) represent pooled data of elective (*n* = 42) and acute (*n* = 40) patients.

**Table 3:** Staff time used in medication processes compared between OSD and TMS, represented as mean values (minutes) with SD per patient.

Staff Time Comparison between OSD and TMS	Combined (*n* = 24)
Simulated total TMS time per patient during hospitalization	42.8 ± 27.1
Total OSD time per patient during hospitalization	30.7 ± 10.9
*Start of OSD, day 1* *per patient*	*12.8 ± 4.35*
*Daily continuation of OSD from day 2 per patient per day*	*5.75 ± 1.66*
Differences in OSD−TMS time per patients during hospitalization	−12.1 ± 8.00
*p*-values, comparison of OSD and TMS	*p* ≤ 0.001

Combined (*n* = 24) represents pooled data of elective (*n* = 11) and acute (*n* = 13) patients.

**Table 4:** Patient perspectives on OSD. Number of respondents (percent) answering on Likert scale questions.

Items	Very Good	Good	Uncertain	Poor	Very Poor	Do Not Know
	(5)	(4)	(3)	(2)	(1)	
Satisfaction with using POM	31 (47)	23 (35)	10 (15)	0 (0)	0 (0)	2 (3)
Satisfaction with bedside locker	42 (64)	18 (27)	4 (6)	2 (3)	0 (0)	0 (0)
Satisfaction with medication in original containers	43 (65)	16 (24)	7 (11)	0 (0)	0 (0)	0 (0)
Satisfaction with responsibility for SMM	39 (59)	19 (29)	8 (12)	0 (0)	0 (0)	0 (0)
Safety with responsibility for SMM	38 (58)	24 (36)	4 (6)	0 (0)	0 (0)	0 (0)
Satisfaction with medication information during hospitalization	35 (53)	21 (32)	6 (9)	1 (2)	0 (0)	3 (4)
Satisfaction with medication information at discharge	35 (53)	20 (30)	4 (6)	1 (2)	0 (0)	6 (9)
Advantage with medication to 10 days at discharge	47 (71)	15 (23)	4 (6)	0 (0)	0 (0)	0 (0)

Combined (*n* = 66) represents pooled data of elective (*n* = 36) and acute (*n* = 30) patients. POM Patients Own Medication, SMM Self-Management of Medication.
